# Supplementary figures and images for: Endocytic protein AP180 assembly domain regulates synaptic vesicle size and release in Caenorhabditis elegans
Source: PLoS Biol. 2026 Feb 6;24(2):e3003643. doi: 10.1371/journal.pbio.3003643 (PMC12893657; doi:10.1371/journal.pbio.3003643)

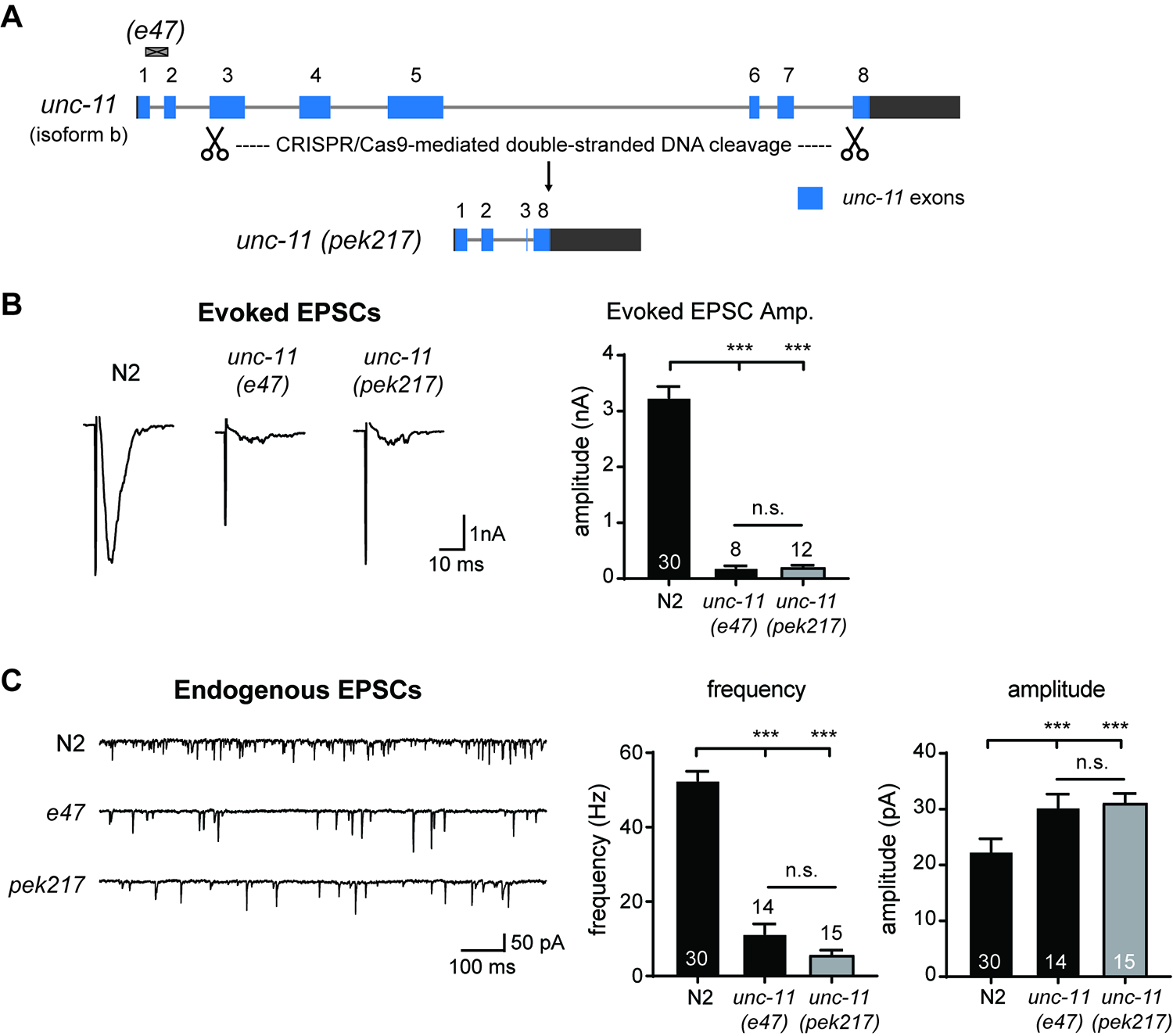

Supplement: S1 Fig — (A) Schematic of the Caenorhabditis elegans ap180 unc-11 gene showing the exons of its isoform b transcript (C32E8.10b.1) and the positions of two mutant alleles. The e47 allele deletes 210 bp across exons 1 and 2 (shaded box). The pek217 allele, generated by CRISPR-Cas9, removes most of unc-11, including part of exon 3, all of exons 4–7, and connecting introns. (B) Representative evoked EPSC traces (left) and summary data for the amplitude of evoked EPSCs (right) are shown. (C) Representative traces (left) and summary data of endogenous EPSC frequency (middle) and amplitude (right) for indicated genotypes. Data are presented as mean ± SEM; the number of worms is indicated in the bar graphs. Error bars represent SEM. Statistical analysis: one-way ANOVA with Tukey’s HSD post hoc test. Significance levels are denoted *** p < 0.001. The data underlying this figure are provided in S8 Data. (TIF) [file pbio.3003643.s001.tif]

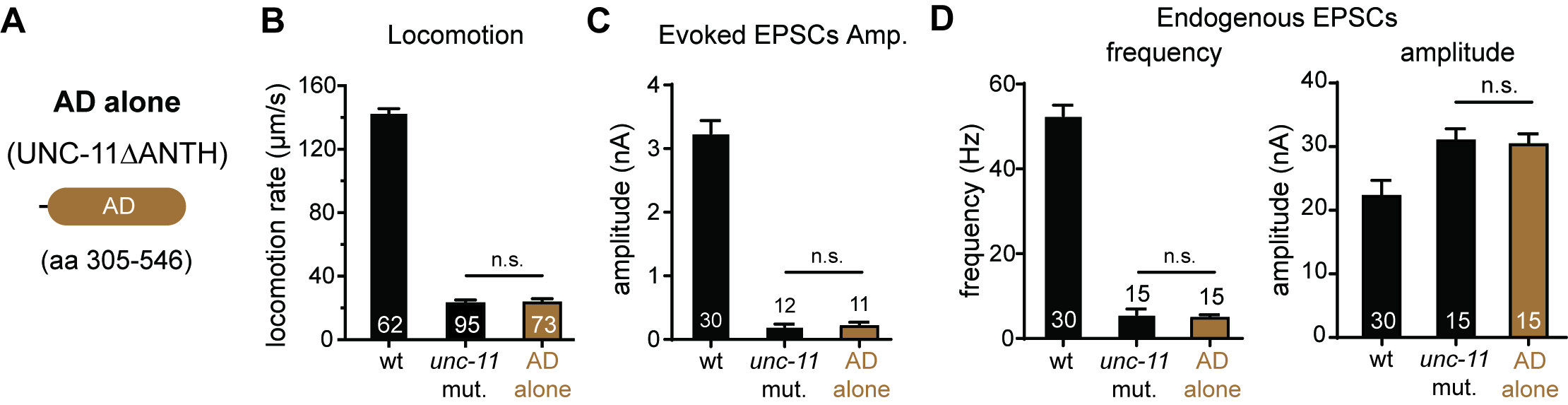

Supplement: S2 Fig — (A) Schematic of UNC-11 AD (residues 305–546) lacking the ANTH domain (residues 1–304). UNC-11 AD is expressed in unc-11 mutant worms via a single-copy transgene under the pan-neuronal snb-1p promoter. “AD” indicates unc-11 mutants expressing UNC-11 AD without ANTH. Summary data for locomotion rates (B), evoked EPSC amplitude (C), and endogenous EPSC frequency and amplitude (D) are shown. The number of worms per genotype is indicated. Data are shown as mean ± SEM. Statistical analysis: one-way ANOVA with Tukey’s HSD post hoc test. “n.s.” indicates no significance. The data underlying this figure are provided in S9 Data. (TIF) [file pbio.3003643.s002.tif]

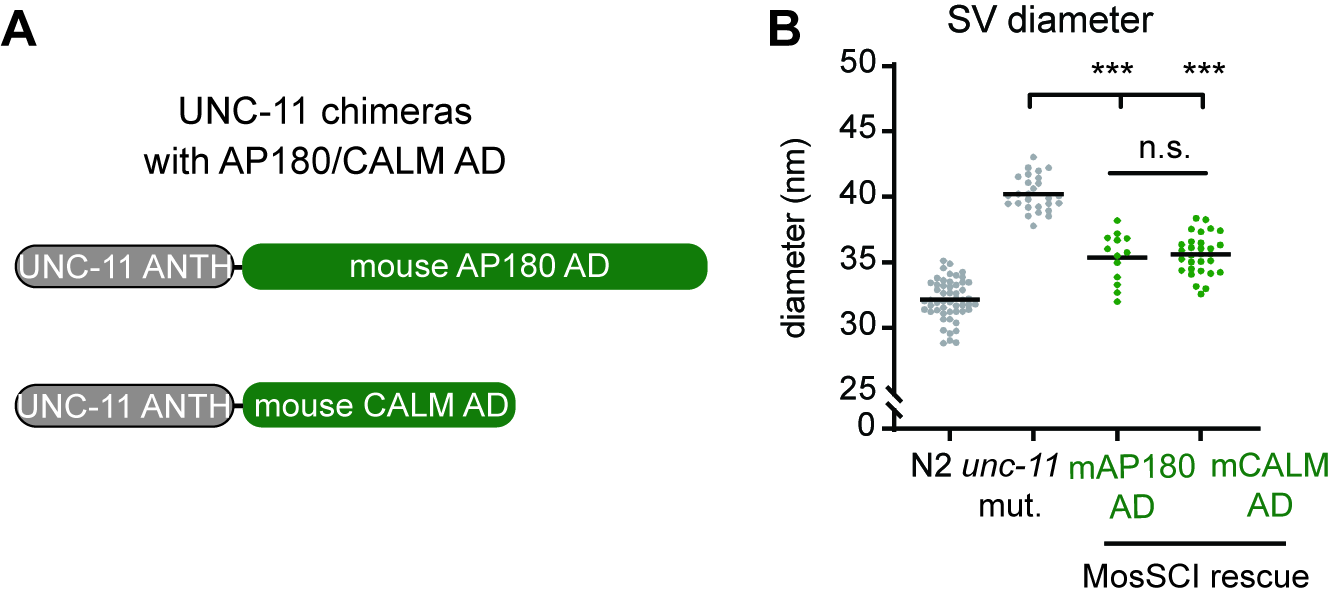

Supplement: S3 Fig — (A) Schematics of chimeric proteins where UNC-11∆AD is fused to the assembly domain of mouse AP180 (mAP180 AD, residues 287–902) or mouse CALM (mCALM AD, residues 287–661). These chimeras are expressed in unc-11 mutant worms via single-copy transgenes under the pan-neuronal snb-1p promoter. “mAP180 AD” and “mCALM AD” refer to unc-11 mutants expressing UNC-11 chimeras containing mAP180 AD and mCALM AD, respectively. (B) Scatter dot plots show synaptic vesicle diameter summary data. Mean values are indicated by the horizontal lines on the graph in the graph. Each data point represents one synaptic profile. “n.s.” indicates no significance. *** p < 0.001 (one-way ANOVA, Tukey’s HSD post hoc test). The data underlying this figure are provided in S10 Data. (TIF) [file pbio.3003643.s003.tif]

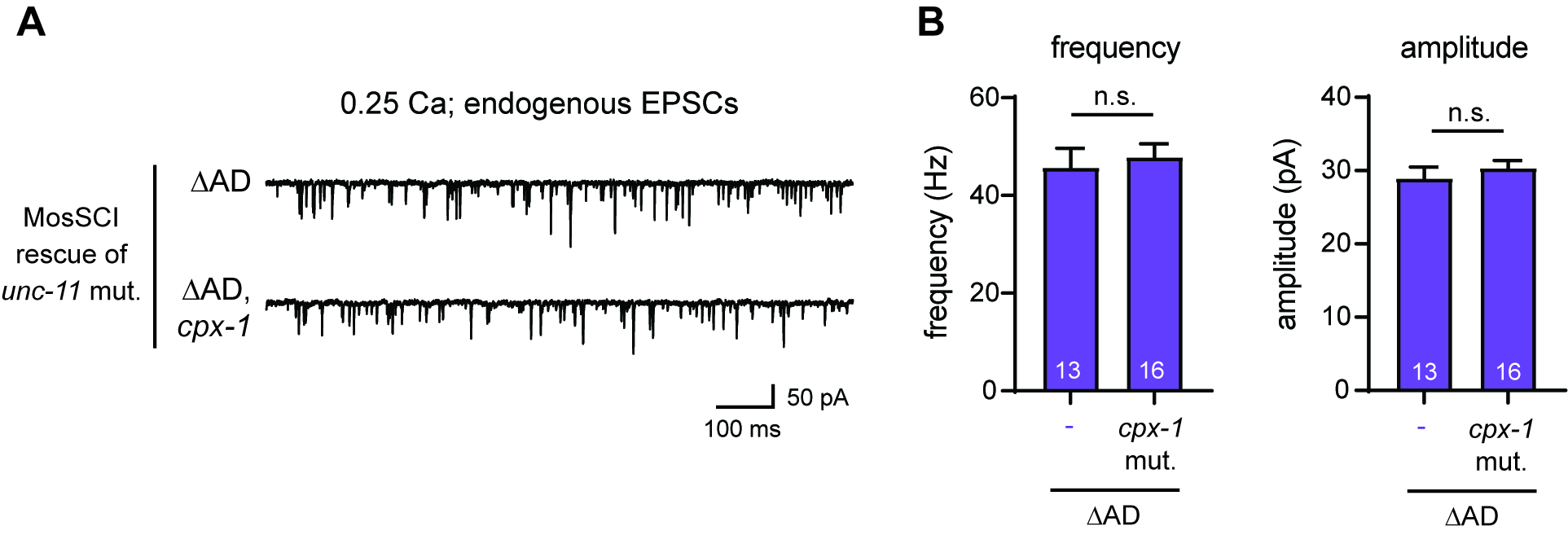

Supplement: S4 Fig — (A) Representative traces and (B) summary data of endogenous EPSC frequency (left) and amplitude (right) for the indicated genotypes. “∆AD”: unc-11 mutant worms expressing UNC-11∆AD in neurons, and “∆AD, cpx-1 mut.”: ∆AD worms also carrying the cpx-1(ok1552) deletion allele. Data are presented as mean ± SEM. Unpaired Student t test with Welch’s test (two-tailed); n.s., not significant. The data underlying this Figure are provided in S11 Data. (TIF) [file pbio.3003643.s004.tif]

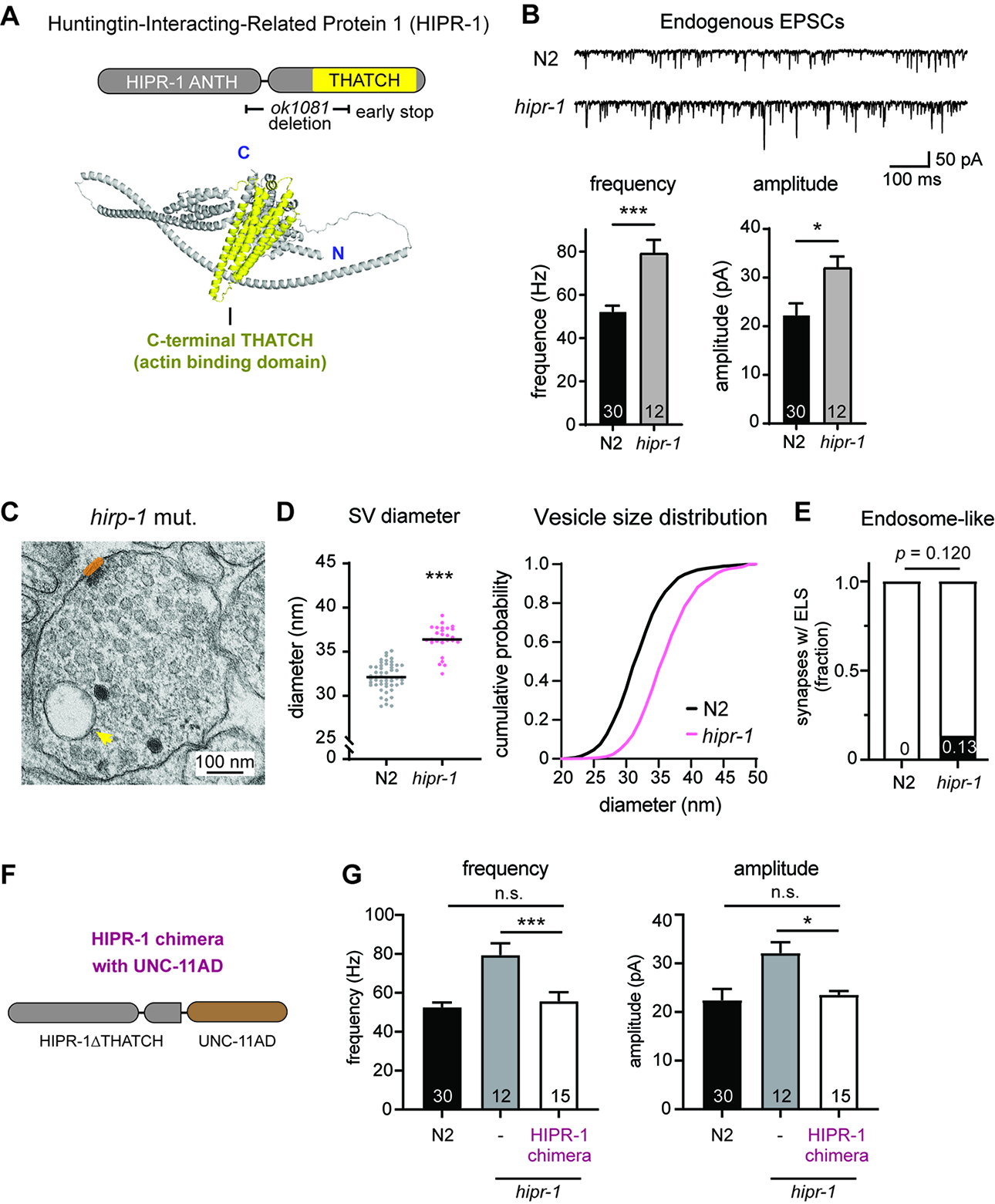

Supplement: S5 Fig — (A) Schematic of the HIPR-1 protein showing the N-terminal ANTH domain and C-terminal actin-binding THATCH domain (yellow). The hipr-1(ok10181) deletion mutation truncates the protein after partially removing the ANTH domain. The predicted AlphaFold structure of HIPR-1 is shown below, with the THATCH motif highlighted in yellow. “N” and “C” indicate termini. (B) Representative traces (top) and summary data (bottom) of endogenous EPSC frequency and amplitude for the indicated genotypes. Data are presented as mean ± SEM and were analyzed by unpaired Student t test; *** p < 0.001; * p < 0.05;. (C) Transmission electron micrograph of a hipr-1 mutant synapse showing dense projections (orange) and an endosome-like structure (yellow arrow). (D) (Left) Summary data of average vesicle diameter per synaptic profile. ***p < 0.001 (unpaired Student t test). Each data point represents one synaptic profile, and mean values are indicated by horizontal lines on the graph. (Right) Cumulative distribution of vesicle diameters for the indicated genotypes. (E) The fraction of synapses containing endosome-like structures (ELS) for the indicated genotypes. Fisher’s exact test was used to analyze categorical variables, and exact p-values are denoted. (F) Schematics of HIPR-1 chimeras. The HIPR-1∆THATCH::AD variant combines HIPR-1 lacking the THATCH domain (deletion of 207 amino acids) with the UNC-11 assembly domain. ∆THATCH::AD was expressed in hipr-1 mutant worms as a single-copy transgene driven by the pan-neuronal snb-1p promoter. (G) Excitatory postsynaptic currents (EPSCs) recorded at the neuromuscular junction. Summary data show endogenous EPSC frequency (left) and amplitude (right). Data are presented as mean ± SEM, with the number of worms analyzed indicated in the bar graphs. Statistical analysis was performed using one-way ANOVA followed by Tukey’s HSD post hoc test. * p < 0.05; *** p < 0.001; n.s., not significant. The data underlying this figure are provided in S12 Data. (TIF [file pbio.3003643.s005.tif]

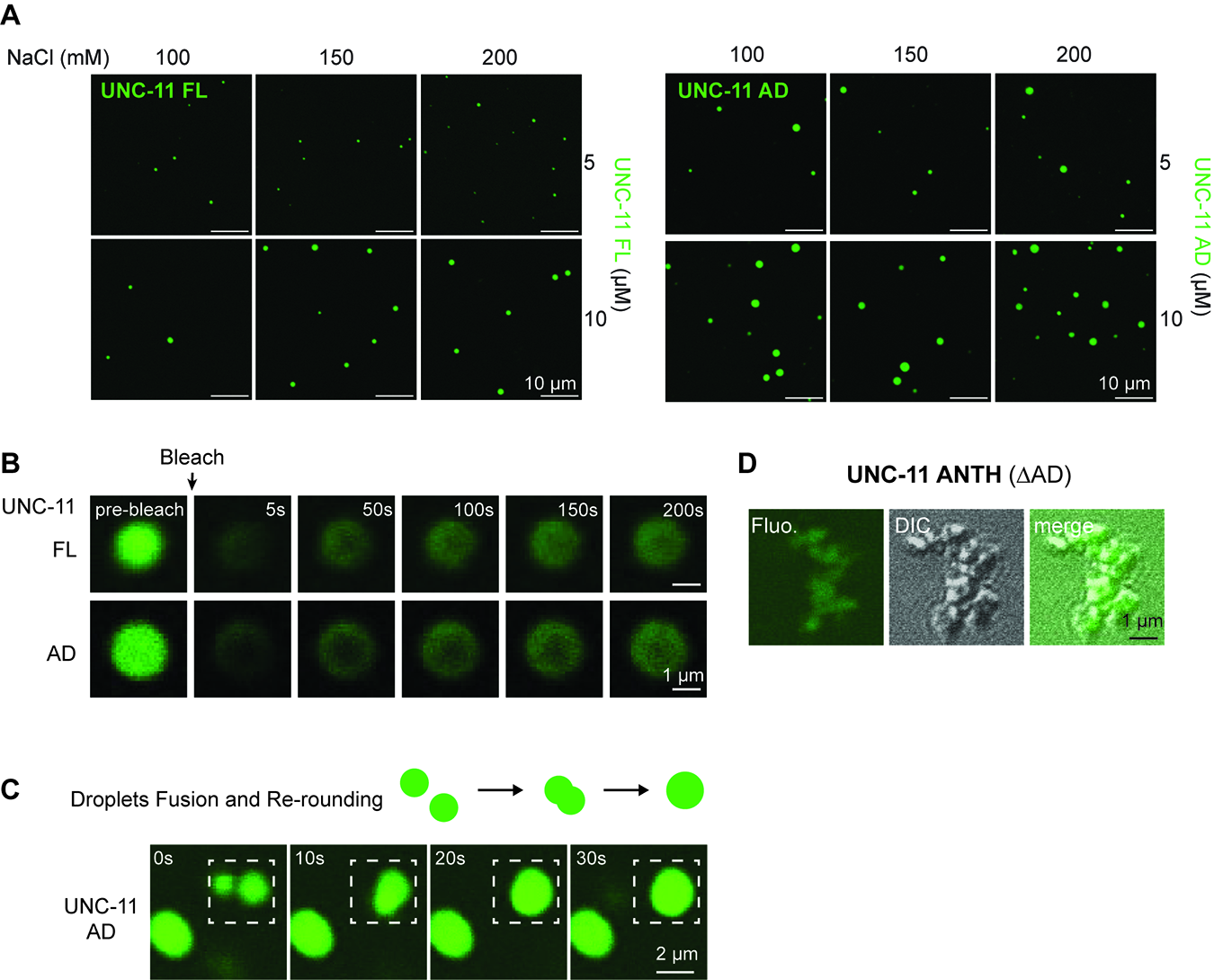

Supplement: S6 Fig — (A) Representative fluorescence images of protein droplets formed by recombinant UNC-11 AD at varying protein concentrations (5 and 10 µM) and ionic strengths (100–200 mM NaCl). The buffer contained 20 mM HEPES (pH 7.7) and 10% w/v PEG. Ten percent of UNC-11 AD was labeled with Alexa Fluor 488 for fluorescence imaging. Scale bar, 10 µm. (B) Fluorescence recovery after photobleaching (FRAP) of condensates formed by full-length UNC-11 (UNC-11 FL) and UNC-11 AD. Proteins (10 µM) were incubated in buffer containing 150 mM NaCl, 20 mM HEPES (pH 7.7), and 10% w/v PEG. FRAP was performed using an Olympus FV1000 confocal microscope with a 60×, 1.4 NA oil-immersion objective (5× zoom). A 488 nm argon laser was used for photobleaching. Scale bar, 1 µm. (C) Fusion and re-rounding of UNC-11 AD condensates (20 µM) in HEPES buffer (200 mM NaCl, 10% w/v PEG, pH 7.7). Scale bar, 2 µm. (D) Fluorescence images showing aggregation of recombinant UNC-11 ANTH (ΔAD) at 10 µM in buffer containing 150 mM NaCl, 20 mM HEPES (pH 7.7), and 10% w/v PEG. Ten percent of UNC-11 ANTH was labeled with Alexa Fluor 488. Scale bar, 10 µm. (TIF) [file pbio.3003643.s006.tif]

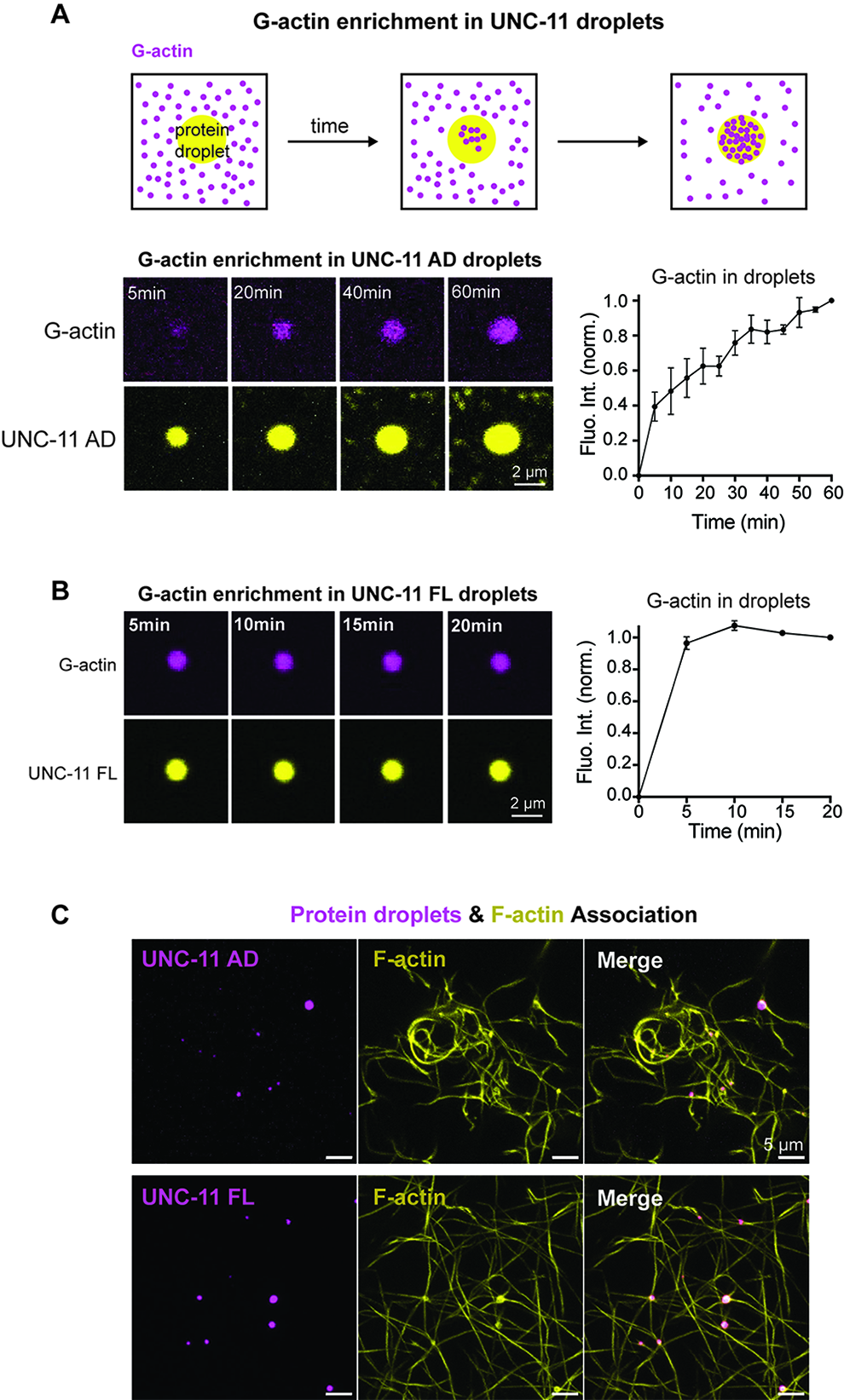

Supplement: S7 Fig — (A) UNC-11 AD condensates enrich monomeric G-actin. (Upper) A schematic cartoon depicts the model in which monomeric G-actin (magenta) is recruited and enriched into protein droplets (yellow). (Lower) Representative fluorescence images of G-actin (50% labeled with Alexa Fluor 488) and UNC-11 AD (20% labeled with Alexa Fluor 405) taken at 5-min intervals for 60 min after adding G-actin (2 µM) to UNC-11 AD (10 µM). Normalized G-actin fluorescence intensity within droplets was quantified over time and plotted (right). Data were collected from four independent experiments and are presented as mean ± SEM. (B) Full-length (FL) UNC-11 condensates also enrich monomeric G-actin. Left: Representative fluorescence images of G-actin (50% labeled with Alexa Fluor 488) and UNC-11 FL (20% labeled with Alexa Fluor 405) taken at 5-minute intervals for 20 min after adding G-actin (2 µM) to UNC-11 AD (10 µM). Right: Normalized G-actin fluorescence intensity within droplets was quantified over time and plotted. Data were collected from four independent experiments and are presented as mean ± SEM. (C) UNC-11 condensates associate with F-actin. Representative fluorescence images show F-actin (Alexa Fluor 488–phalloidin, yellow) and protein condensates of UNC-11 AD (top) or UNC-11 FL (bottom, magenta). F-actin was assembled from 2 µM G-actin followed by addition of UNC-11 variants (10 µM). Scale bar, 5 µm. The data underlying this figure are provided in S13 Data. (TIF) [file pbio.3003643.s007.tif]

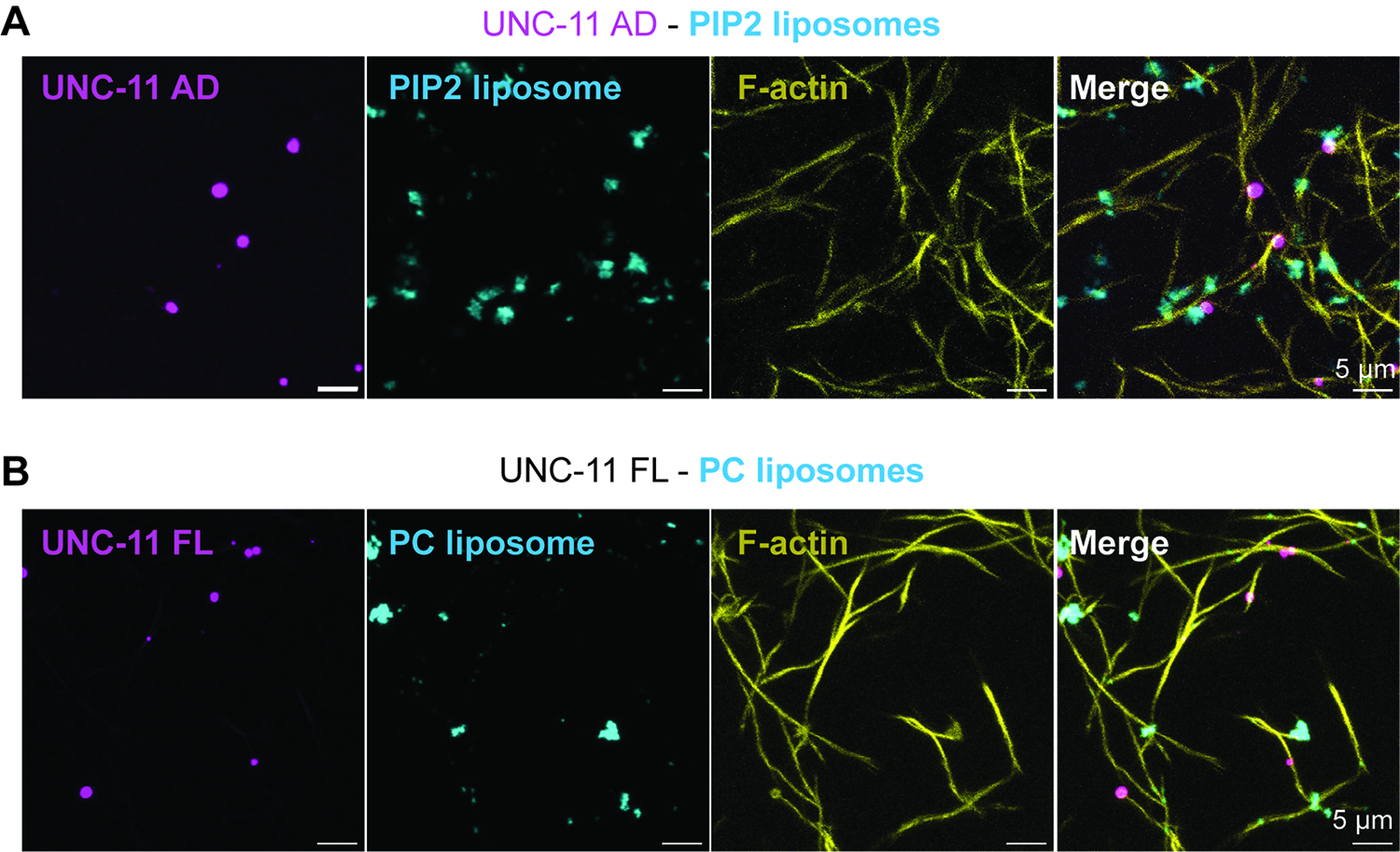

Supplement: S8 Fig — (A) Representative fluorescence images of samples containing F-actin, PIP2(2%) liposomes, and UNC-11 AD that lacks the membrane-binding ANTH domain. UNC-11 AD is labeled with Alexa Fluor 405 (magenta), F-actin with Alexa Fluor 488-phalloidin (yellow), and liposomes with Rhodamine PE (2%, cyan). Liposomes contained 500 µM total lipids (2% PIP2, 25% PS, 71% PC, and 2% Rhodamine-PE). Scale bar, 5 µm. (B) Representative fluorescence images of samples with F-actin, PC liposomes (98%PC, 2%Rhodamine-PE), and UNC-11 FL. UNC-11 binds liposomes carrying anionic phospholipid but not PC-only liposomes. UNC-11 FL was labeled with Pacific Blue (magenta), F-actin with Alexa Fluor 488-phalloidin (yellow), and liposomes with Rhodamine PE (2%, cyan). Liposomes contained 500 µM total lipids (98% PC and 2% Rhodamine-PE). Scale bar, 5 µm. The data quantified from the images in this figure are presented in Fig 7, and the source data are provided in S7 Data. (TIF) [file pbio.3003643.s008.tif]
