# Supplementary material for: Endocytic protein AP180 assembly domain regulates synaptic vesicle size and release in Caenorhabditis elegans
Source: PLoS Biol. 2026 Feb 6;24(2):e3003643. doi: 10.1371/journal.pbio.3003643 (PMC12893657; doi:10.1371/journal.pbio.3003643)
Supplement: Attachment — Submitted filename: Response to Reviewers_yu.docx [file pbio.3003643.s022.docx]

**Response to Reviewers** (PBIOLOGY-D-25-01789R1)

We appreciate the thoughtful evaluation of our manuscript by the reviewers and the editor. We are encouraged by their recognition of the importance of our study and have carefully considered all the points raised. In response, we have added new data and revised the text to address the concerns and improve the clarity of the manuscript. Our detailed responses to the reviewers’ comments are provided below.

**Reviewer #1**

Major critique

1) The authors present a simple linear model for the inhibitory function of the AD domain: the AD decreases vesicle diameter and large vesicles evade inhibition of fusion by complexin. In support of this model, a double mutant between AD deletion and complexin has the same elevated mini frequency as either single mutant. However, beyond this observation the single mutants do not phenocopy each other. Specifically, complexin mutants have decreased synaptic vesicle number and dramatically decreased evoked responses. However, deletion of AD results in no significant decrease in synaptic vesicle number and an increase in evoked response. Thus, the increase in synaptic transmission seen in AD deletion is unlikely to be explained simply as large vesicles evading regulation by complexin, otherwise the mutants should look more like complexin mutants. These caveats should be explicitly spelled out in the text.

We thank the reviewer for this thoughtful comment, which prompted us to test a key prediction of our model that had not been examined in the original manuscript. The reviewer correctly points out that complexin (Cpx) regulates two distinct aspects of synaptic transmission: clamping endogenous mini release and facilitating evoked release. In our original manuscript, we reported only endogenous EPSC frequency. The reviewer's comments led us to test evoked responses in *unc-11∆AD* worms with and without *cpx-1*.

Previous studies have shown that these two Cpx functions depend on distinct structural elements. The curvature-sensing C-terminal domain (CTD) is required for clamping spontaneous release but not for facilitating evoked release. Lottermoser et al. (PMID: 40321081) demonstrated that a complexin variant lacking the curvature-sensing CTD (CPX∆CTD) fails to suppress mini rates (Figure 1F-G), indicating loss of the clamping function when curvature-sensing is disrupted. However, evoked EPSC amplitudes were largely restored regardless of whether the CTD was present (Figure 1J, columns 3-4). Together, these findings indicate that the curvature-sensing CTD is essential for clamping mini release but dispensable for Cpx's facilitation of evoked release, while both require SNARE-binding regions of the protein (e.g., Martin et al., PMID: 21215634).

Based on this dual-function framework of complexin, our model predicts that enlarged synaptic vesicles in *unc-11∆AD* mutants will evade curvature-dependent clamping because their increased size mismatches the curvature-sensing CTD, yet they will retain curvature-independent facilitation. Thus, *unc-11∆AD* single mutants should display elevated endogenous mini release due to loss of curvature-dependent clamping, while evoked release should remain intact or even enhanced because curvature-independent Cpx facilitation remains functional. In contrast, *unc-11∆AD; cpx-1* double mutants should show increased endogenous mini release similar to *unc-11∆AD* single mutants (both lack functional Cpx clamping) but reduced evoked release similar to *cpx-1* null mutants, reflecting the complete loss of complexin-mediated facilitation.

Our new data confirm these predictions: evoked response amplitudes in *unc-11∆AD; cpx-1* double mutants decrease to levels comparable to *cpx-1* null mutants alone (Figure 4B). This result demonstrates that while enlarged vesicles at *unc-11∆AD* synapses evade curvature-dependent inhibition by *cpx-1*, they remain fully dependent on curvature-independent facilitation by *cpx-1* for evoked release.

In summary, we agree with the reviewer that *unc-11∆AD* synapses do not phenocopy *cpx-1* null synapses, and our model explicitly predicts this outcome. In *unc-11∆AD* mutants, endogenous release increases because curvature-dependent clamping is impaired, whereas evoked release remains intact owing to preserved SNARE-dependent facilitation. When *cpx-1* is removed from *unc-11∆AD* mutants, both mechanisms fail, leading to reduced evoked responses.

In the revised text, we have incorporated these new data, clarified the dual roles of complexin, and explained how the distinct phenotypes of *unc-11∆AD* and *cpx-1* mutants are consistent with and reinforce our model.

*Revision to the manuscript:*

Lines 189-190: Revised subtitle – “Enlarged synaptic vesicles at UNC-11∆AD synapses evade complexin-mediated suppression of endogenous EPSCs.”

Lines 193-196“One such mechanism involves complexin (CPX-1), a presynaptic protein that suppresses endogenous EPSCs in *C. elegans* through SNARE binding and curvature sensing (51-53), yet promotes evoked release through curvature-independent, SNARE-dependent mechanisms (52, 53).”

Lines 202-205: “In contrast, evoked EPSC amplitudes are significantly reduced in *unc-11∆AD; cpx-1* double mutants to levels comparable to *cpx-1* null mutants (Figure 4B), indicating that enlarged vesicles remain dependent on the curvature-independent facilitation of evoked release by CPX-1.”

Lines 220-223: “Collectively, these findings suggest that enlarged synaptic vesicles at UNC-11∆AD synapses escape CPX-1 inhibition of endogenous EPSC because their altered size disrupts curvature-dependent CPX-1 recruitment, while evoked release continues to rely on curvature-independent, SNARE-dependent facilitation.”

Line 483-484 (revised title of Figure 4): “Complexin inhibition of vesicle fusion is impaired at UNC-11∆AD synapses but remains essential for evoked release.”

Lines 488-489 (updated legend of Figure 4): “**(B)** Representative traces of evoked EPSCs (*left*) and summary data for evoked EPSC amplitude (*right*) are shown.”

Figure 4**:** Updated to include **(B)** evoked EPSC data for N2, ∆AD, *cpx-1* mut., and *cpx-1* mut.; ∆AD worms.

2) Elevations in mini frequency at calcium levels over 1mM can be challenging to measure.  It is possible that the lack of an increase in the cpx-1 unc-11(ΔAD) double mutant is the result of a ceiling effect; in other words, at 1mM calcium complexin mutants are already fusing at their maximum rate and can't be further increased. Adding an additional data point at 0.25mM or 0.5mM external calcium would help support the model presented.

As suggested by the reviewer, we performed additional electrophysiological recordings at 0.25 mM external Ca^2+^. Consistent with our previous findings at 1 mM Ca^2+^, deletion of *cpx-1* did not further increase release frequency at UNC-11∆AD synapses (Supplemental Figure 4). These results indicate that the inhibitory function of CPX-1 is already compromised when vesicles are enlarged, rather than limited by a ceiling effect.

*Revision to the manuscript:*

Lines 200-202: “However, at UNC-11∆AD synapses, *cpx-1* deletion has no effect on either frequency or amplitude at 1 and 0.25 mM external Ca^2+^ (Fig. 4A and Supplemental Fig.4A), suggesting that the inhibitory function of CPX-1 is already compromised when vesicles are enlarged.

Lines 742-744 (updated methods): “The extracellular recording solution contained (in mM): 150 NaCl, 5 KCl, 1 CaCl_2_ (or 0.25), 5 MgCl_2_, 10 glucose, and 10 HEPES, adjusted to pH 7.3 with NaOH and to 330 mOsm with sucrose.”

Added new Supplemental Figure 4, updated to include electrophysiology data collected at 0.25 mM Ca^2+^.

Lines 581-586 (updated title of Supplemental Figure 4): “Synaptic transmission at UNC-11∆AD synapses is insensitive to *cpx-1* mutation at 0.25 mM external calcium.”

3) The experiments supporting the interaction with actin as key to AP180 function are: First, deleting the actin-binding THATCH domain from the HIPR-1 portion of the UNC-11::HIPR-1 chimera abrogates rescue. Second, replacing the AD of UNC-11 with an actin-binding domain results in a fully functional UNC-11 protein. There are two possible explanations: either UNC-11 binds actin by binding HIPR-1, or the AD is itself an actin binding module. Two experiments could further tease which model is correct. 1) The UNC‑11(ΔAD)::Lifeact construct could be put into a hipr-1 mutant background. 2) a hipr-1 mutant with hipr-1(ΔTHATCH)::UNC-11-AD could be tested for rescue. The first experiment would test if UNC-11s role in actin binding is simply via HIPR-1. If this rescues, the AD domain most likely functions to recruit HIPR-1 which in turn binds actin. The second experiment would test the novel idea that UNC-11 itself can bind actin. Of course, there are numerous reasons that these experiments might not work, but success in either of them would go a long way towards establishing the details of link between UNC-11 and actin. Adding them would strengthen the manuscript. Absent new experiments, we suggest a thoughtful explanation of the caveats of the current experiments. As currently written it's easy for the reader to be misled into thinking that UNC-11 interacts directly with actin - a point not supported by the experiments.

We thank the reviewer for this suggestion. The reviewer correctly notes that our original data support a functional connection between UNC-11 and actin but do not directly distinguish whether UNC-11 binds actin through HIPR-1 recruitment or via direct actin interaction. Prompted by this comment, we carried out both genetic and biochemical experiments to test for direct UNC-11-actin interactions.

First, as the reviewer suggested, we expressed a chimeric HIPR-1 protein in which the THATCH domain was replaced by the UNC-11 AD domain (HIPR-1∆THATCH::AD) in *hipr-1* mutant neurons (Supplemental Fig. 5F). This chimera returned both the frequency and amplitude of endogenous EPSCs to wild type levels (Supplemental Fig. 5G). These results demonstrate that the UNC-11 AD domain can functionally substitute for the actin-binding HIPR-1 THATCH domain *in vivo*, supporting the model that they share a common mechanistic role in linking endocytosis to the actin cytoskeleton.

Second, we performed biochemical and imaging studies using recombinant full-length UNC-11, UNC-11 AD, and UNC-11 ANTH(∆AD) to test for actin interactions. Like many intrinsically disordered protein domains, both full-length UNC-11 and the UNC-11 AD domain undergo liquid-liquid phase separation (Supplemental Fig. 6), whereas the compactly folded ANTH domain alone does not. Importantly, both full-length UNC-11 and UNC-11 AD condensates enrich monomeric actin (Supplemental Fig. 7A), accelerate actin polymerization (Fig. 7A), and bind assembled actin filaments (Supplemental Fig. 7B), providing biochemical evidence for UNC-11 AD-actin interactions.

We further tested whether full-length UNC-11 can simultaneously engage both membranes and actin through its PIP2-binding ANTH domain and actin-interacting AD domain. Full-length UNC-11 droplets localized preferentially to the interface between PIP2-containing liposomes and actin filaments compared to UNC-11 AD lacking the membrane-binding ANTH domain (Fig. 7B-C and Supplemental Fig. 8A). This localization depends on specific lipid interactions, as full-length UNC-11 droplets failed to localize to the liposome-actin interface when PC liposomes lacking PIP2 were used (Fig. 7C and Supplemental Fig. 8B).

Together, these genetic and biochemical results demonstrate that UNC-11 directly interacts with actin and can bridge membranes to the actin cytoskeleton. In the revised manuscript, we have clarified the model and incorporated these new data to establish the molecular basis of the role of UNC-11 in engaging actin.

*Revision to the manuscript:*

Line 290-296: “To test whether the UNC-11 AD domain can functionally substitute for the HIPR-1 THATCH domain *in vivo*, we expressed a chimeric HIPR-1 protein in which the THATCH domain was replaced by UNC-11 AD (HIPR-1∆THATCH::AD) in *hipr-1* mutant neurons (Supplemental Figure 5F). This chimera restored endogenous EPSC frequency (56 ± 5 Hz) and amplitude (23.5 ± 1 pA) to wild-type levels (Supplemental Figure 5G), indicating that the UNC-11 AD domain can replace the actin-binding HIPR-1 THATCH domain *in vivo*, supporting a model in which both domains share a common mechanistic role in linking endocytosis to the actin cytoskeleton.”

Lines 298-326: Added a new section in Results titled “UNC-11 condensates couple actin assembly to PIP2-rich membranes,” incorporating newly obtained biochemical and imaging data.

Lines 405-425: Added a new section in Discussion titled “AP180 condensates interact with actin,” summarizing genetic and biochemical evidence that AP180 links actin to endocytic membranes.

Lines 532-548 (updated Figure 7 legend): “UNC-11 condensates support actin polymerization and bridge F-actin to PIP2-containing membranes.”

Lines 588-589 and 601-608 (revised legend of Supplemental Figure 5): Updated title and added new description for panels (F-G).

Lines 610-651: Added legends for Supplemental Figures 6-8.

The strain list and Methods sections have been updated to include new strains and information.

Minor critique and typographical errors

1) The isoform of unc-11 being used is never mentioned (even in supplemental Figure 1). The gene structure looks like isoform b. Please indicate which isoform is shown in Fig S1.

The isoform used in this study is *unc-11b*. Supplemental Figure 1A and its legend have been updated to indicate that the gene structure shown corresponds to isoform b (Line 551 in the revised manuscript).

2) There is a general lack of consistency with mentioning statistical significance in the text (e.g lines 132 and 163 could say "NS"). In keeping with this, often a comparison is made in the text but no accompanying significance bar is present in the referenced graph (e.g. line 234 compares UNC-11∆AD::NfM-CD to unc-11 but this is not highlighted on graph). Significance indicators can make a graph busy, so I understand why the author omits some comparisons, but don't quite understand why others are included but not referenced. I would rather the author add omitted but referenced significance bars, rather than remove included but unreferenced bars, because in the future, someone may appreciate the comparison, even if it was not needed to support the story presented in this text.

We have revised both the figures and text to ensure consistent reporting of statistical significance. “Not significant” has been added where appropriate, and missing significance bars have been included in the relevant figures, including the comparison between UNC-11∆AD::NfM-CD and *unc-11* in Figure 5C.

3) FIGURE 3C and FIGURE S5D.  The distribution of SV diameters could be more clearly presented.  Currently the Y-axis is the total number of vesicles, which simply reflects the number of N2, unc‑11 and delta-D profiles counted.  The data should be normalized so the y-axis is 'fraction of total vesicles" in each bin.  Moreover, the histograms are overlapping and occlude the other distributions. Making them transparent or "skylines" would facilitate comparisons.  A better alternative is to show the size distribution as a cumulative plot rather than binning the data, so that every data point is shown.

As suggested by the reviewer, we have replaced the histograms in Figure 3C and Supplemental Figure 5D with cumulative distribution plots of synaptic vesicle diameters to provide clearer and more direct comparisons across genotypes.

4) Mini amplitudes.  One might also consider a cumulative plot for mini amplitudes, which is a parallel readout for the synaptic vesicle diameter - the shape of the cumulative plots would be similar.

We have added a cumulative distribution plot of miniature EPSC amplitudes to Figure 1G. UNC-11∆AD worms display a right-shifted cumulative distribution of endogenous EPSC amplitudes compared with wild-type synapses.

5) Be consistent between "supplemental" and "supplementary" when referring to supplemental figures.

We have standardized this to “Supplemental” throughout the revised manuscript.

6) Line 90: "RESULT" should read "RESULTS"

Corrected in the revised manuscript.

7) Line 103: "Supplemental Figure 1B-C" might just refer to "1C".

Corrected in the revised manuscript. (Line 102)

8) Lines 130-131: "UNC-11∆AD: 37 ± 2 pA; unc-11 mutant: 31 ± 2 pA; not significant; Figure 1E". This specific comparison is not highlighted with a significance value on graph, whereas others are indicated but not mentioned in the text.

“Not significant” has been added to the revised Figure 1F, and the updated figures in revised manuscript now include the corresponding significance indicators.

9) Lines 174-175: full-length UNC-11 data should be in Figure 3, not supplemental. It would only add one group to the scatter plot and only one micrograph picture.

We have now included these data in the revised Figure 3, as suggested by the reviewer.

10) Line 212: "…C-terminal helix (ΔCH; Figure 4C)". Did you mean "Central Helix"? This is often referred to as the CH, and is responsible for SNARE binding. There is a C-terminal amphipathic helix, but this is thought to interact with membrane.

Corrected in the revised manuscript. (Line 217)

11) Line 271: "Figure 6 lower" should read "Figure 6a lower"

Corrected in the revised manuscript. (Line 279).

12) Line 316: "The AD is intrinsic disorder" should read "The AD is intrinsically disordered"

Corrected in the revised manuscript (Line 383).

13) Lines 375-376: "Mean values are indicated in the graph" but don't seem to be.

We have revised the figure legends for clarity to state, “Mean values are indicated by horizontal lines on the graph.” (Line 475)

14) Figure 3b: are the numbers indicated in bar chart the number of profiles counted?

Yes. We have clarified this in the revised Figure 3 legend as: “Bar graph summarizing the number of vesicles per synaptic profile, with the numbers of synaptic profiles analyzed indicated on the bars.” Lines: 476-477.

15) Figure 5d: half of chart is redundant with 3d.

The N2 and *unc-11* data are presented again as controls to facilitate comparison with the experimental conditions.

16) Figure 6a: include label for grey portion of unc11deltaAD.

The label for the grey portion of unc-11∆AD has been added, as suggested by the reviewer.

17) Figure 6c: consider showing statistics for lifeact to N2 comparison.

The statistical comparison between Lifeact and N2 has been added in the revised Figure 6C.

18) Supplemental Figure 4a: add label to unc-11 ANTH.

Label for UNC-11 ANTH added as suggested.

19) Supplemental Figure 5a: Here color is used to highlight the THATCH domain. In Figure 1a the default color indicates confidence probably to highlight that is it disordered, but are not indicated in Figure 1a.  I don't mind, but consider being consistent.

We revised the Figure 1A legend to state that the color scale represents per-residue model confidence (predicted Local Distance Difference Test score; dark blue: very high; light blue: high; champagne: moderate; brown: low). Lines: 441-443

20) Supplemental Figure 5d: like Figure 3c, make y-axis "fraction of vesicles" to normalize the data.

As noted above, Supplemental Figure 5D and Figure 3C have been revised to show cumulative distributions.

21) Supplemental Figure 5e: instead of "EL per synapse" like 3d and 5d, make this "fraction of synapses with ELS". This is what the figure legend suggests you are presenting.

Supplemental Figure 5E has been updated as suggested.

**Reviewer #2:**

There are a few minor points that would benefit from clarification.  In the complexin experiment (Fig. 4D), it would help to know that rescue with WT does not work.  Clearly, the endogenous complexin does not rescue but it would help to show that the introduced complexin also does not work in the absence of the UNC11 AD.  The text describing Fig. S4 should also be revised:  UNC11ΔAD was not replaced; rather, the AD from AP180 was added.

Following the reviewer’s suggestion, we overexpressed wild-type complexin in neurons carrying UNC-11∆AD. CPX-1 overexpression did not restore the elevated frequency of endogenous EPSCs, indicating that additional wild-type complexin does not suppress vesicle release at UNC-11∆AD synapses. This supports the conclusion that the defect arises from impaired engagement of CPX-1 with enlarged vesicles rather than insufficient protein levels. These new data are presented in Figure 4D.

The text describing Supplemental Figure 4 has been revised as suggested.

*Revision to the manuscript:*

Lines 211-212: In contrast, overexpression of wild-type CPX-1 failed to reduce the elevated mini frequency (Figure 4E).

Lines 182-183: Revised text “we replaced UNC-11 AD with the AD from mouse AP180 (mAP180 AD) or its homolog CALM (clathrin assembly lymphoid myeloid leukemia”.

However, the central question is still whether the effect on vesicle size simply correlates with the increased release or causes it.  The various rescuing constructs all fix both size and release but maybe these are independent effects.  Alternatively, altered vesicle composition might account for the changes in release.  Loss of AP180 impairs VAMP2 trafficking but what does the assembly mutant do to VAMP2 or other vesicle proteins?   It would also help to show that other mutations affecting SV size (affecting other aspects of the endocytic machinery) all have the same effect.  Additional evidence to support a causal role for the effect of vesicle size on release would strengthen this excellent study.  I support publication in PLOS Biology but with additional data (or consideration if evidence already exists) that addresses this question.

We thank the reviewer for supporting publication of our manuscript and for this thoughtful question, which highlights the challenge of disentangling vesicle morphology from protein composition in controlling release properties. The two are closely linked, and it is not straightforward to separate their effects. A definitive test would perhaps require isolating synaptic vesicles from *ap180∆AD* or *hipr-1* mutant synapses and performing quantitative proteomic analysis, which is an important direction for the future.

Still, several lines of evidence in this study suggest that vesicle morphology itself has a direct impact on release. First, HIPR-1 provides an independent test case because, unlike AP180, it is not a cargo adaptor. Loss of HIPR-1 results in enlarged vesicles and elevated release frequency, and both are rescued by restoring actin binding. This parallel with UNC-11∆AD indicates that the shared driver is actin-dependent morphological control rather than changes in cargo sorting.

Second, the complexin experiments reveal a specific link between vesicle curvature and release regulation. At *ap180∆AD* synapses, complexin's curvature-dependent clamping of spontaneous release is selectively impaired, while its SNARE-dependent facilitation of evoked release remains intact. Tethering CPX-1 directly to synaptic vesicles restores its inhibitory function. These results are more consistent with a curvature mismatch between complexin and enlarged vesicles than with changes in protein composition.

Third, the Lifeact rescue provides a genetic separation of morphology from composition. Adding the small actin-binding Lifeact peptide to UNC-11∆AD restores both vesicle size and release frequency. Because Lifeact contributes only actin binding and does not restore cargo sorting, this result supports a direct role for actin-dependent size control in regulating vesicle release.

Some evidence also exists in the literature. For example, synaptophysin/synaptogyrin quadruple knockout mice display enlarged synaptic vesicles and elevated release probability with increased quantal size (Figure 2 in Raja, 2019 eLife PMCID: PMC6519982), consistent with our observations. In addition, reconstituted fusion assays comparing purified synaptic, chromaffin, and insulin vesicles revealed that calcium-triggered fusion time constants increase with curvature, correlating with cellular release rates (Kreutzberger et al, 2019 Nat Commun., PMCID: PMC6715626).

Together, the convergence of HIPR-1 and AP180 findings, selective loss of curvature-dependent complexin clamping, rescue by minimal actin-binding motifs, and existing evidence in the literature supports a model in which vesicle size influences release through curvature-sensing mechanisms.

We have also expanded the Discussion to acknowledge that vesicle morphology and composition likely act together to influence release:

Lines 364-377: "It is plausible that increased fusogenicity results not solely from altered vesicle morphology, but also from changes in vesicle protein networks. For example, AP180 participates in VAMP2 sorting during endocytosis (28, 29, 33, 34, 38, 71, 72). However, several lines of evidence suggest that morphological control is a primary driver of the release phenotype. In support of this, loss of HIPR-1, which unlike AP180 is not an adaptor for vesicle protein sorting, produces similar increases in vesicle size and release frequency. Both the enlarged vesicle phenotype and elevated release frequency are rescued by restoring actin interactions, indicating that mechanisms beyond vesicle protein sorting contribute substantially to enhanced release probability. Additionally, the selective loss of curvature-dependent complexin function at UNC-11∆AD synapses, while SNARE-dependent functions remain intact, is more consistent with a geometric mismatch than with altered protein composition. While the convergence of genetic evidence strongly supports vesicle morphology as a key determinant of release properties, fully establishing causality without confounding influences from vesicle composition would likely require biochemical isolation of synaptic vesicles from *ap180∆AD* or *hipr-1* mutant synapses followed by quantitative proteomic analysis."

We thank the reviewer again for their support and constructive feedback.
