# Supplementary material for: Endocytic protein AP180 assembly domain regulates synaptic vesicle size and release in Caenorhabditis elegans
Source: PLoS Biol. 2026 Feb 6;24(2):e3003643. doi: 10.1371/journal.pbio.3003643 (PMC12893657; doi:10.1371/journal.pbio.3003643)
Supplement: Attachment — Submitted filename: _Response to Reviewers.docx [file pbio.3003643.s023.docx]

**Response to Reviewers** (PBIOLOGY-D-25-01789R3)

We thank the reviewers for their careful evaluation and constructive feedback throughout the review process. Below, we address the remaining comments.

**Reviewer #1**

**Comment:** Please include a reference to Snead et al., 2014 when stating that the complexin C-terminal domain is curvature sensing.

**Response:** We have added a citation to Snead et al., 2014 (Ref. 70) in the Introduction.

**Reviewer #2**

**Comment:** Why does complete loss of *unc-11* not increase spontaneous release frequency despite enlarged synaptic vesicles?

**Response:** We now address this point explicitly in the Discussion. The revised text states: “This size-dependent modulation of basal release is not observed in the *unc-11/ap180 null* mutant. Although vesicles are similarly enlarged in this background, loss of the AP180 ANTH domain disrupts SNB-1/VAMP2 sorting and recycling to synaptic vesicles. The resulting depletion of vesicle-associated SNAREs substantially decreases fusion competence, thereby masking the increased fusion probability expected from vesicle enlargement.” (Lines 361 to 365)
